# Medical Miscellanies

**Published:** 1818-04

**Authors:** 


### PART IV.

### MEDICAL MISCELLANIES.

Quicquid agunt Homines.

*Dr. John Walker, in his "Reply, &c." just published, has drawn up a short Biographical Account of the late Dr. Woodville, in Vindication of the Character of that deceased Physician, which Dr. W. conceives to have been unjustly attacked in the "History of Vaccination." We think it may not be unacceptable to our readers if we present them both with the Charge and the Vindication, in the Doctor's own Words.*

"Here had I laid down my pen on the Reply to James Moore; but as the precept, '*Nil nisi bonum de mortuis,*'

has not restrained the very learned author from calumniating the memory of the deceased, as well as the ways of the living, I must offer a little of the biography of the author of Medical Botany, and principal Founder of Vaccination in the metropolis, whence it has been so happily diffused through every quarter of the world.

"The following is the scleratic attack on the memory of Dr. Woodville:

"J. M. In a few years he fell a victim to intoxicating liquors, swallowed, as was believed, to alleviate a fixed melancholy, which proceeded from an unhappy deed perpetrated in early life.

"*Biography of Dr. Woodville.*

"J. W. From the public Grammar School of our native town (Cockermouth), Woodville, a young Quaker, had just been removed, and placed behind the counter of an apothecary, on the side of the street directly opposite my father's house; when, having learnt to read and spell, and say the *long* assembly's catechism by rote, I was sent to the same school, at six years of age, to learn the more easily comprehensible rules of Lily on a dead language. He had several coeval apprentices in the town, of the same profession; and soon, I remember well, he was not too conceited to appeal to his little neighbour school-boy, in disputing with his brother Esculapianinos on the rules of grammar. But, is not this, à la Moore, bringing forward the puff-ball? Let us pass on then to the man's estate of the author of the Medical Botany. He had graduated, travelled on the continent, and returned to his native home; when, residing at his mother's, at a neighbouring village, after midnight, the rest of the family being gone to bed, he heard the tread and low toned voices of men hovering about the premises, who seemed to take their stand by the window of the room in which he was sitting. On his calling out to them they made no answer; but, on his going to the kitchen with his light, to see if all was safe there, a fowling piece loaded with shot in his hand, one of them coming up to the window, which looked into a private yard, he renewed his call to them, when moving forwards and backwards without making any answer, they appeared evidently reconnoitering. Coming again close up to the window, he called out, 'If you don't be gone I'll shoot you;' and on presenting the piece, he actually broke the window with the muzzle of it, and in the agitation of the moment involuntarily pulled the trigger, when a poor innocent youth immediately fell. He was related to a maid servant in the house, and had accompanied his friend, another young man, a soldier, their first cousin, by appointment, to take his leave of her previous to his setting out for the army the next morning. These worthy young men had taken a cheerful glass with some friends in the town, and not been punctual to the appointment. They were so much past the time, that the young woman had given them up, and gone to bed.

A medical character, who can, as well as Dr. Jenner, sport on his finger a brilliant mark of Imperial favour, the Assistant Director of the Royal Jennerian Society, had, in his History of Vaccination (in which the name of so great a character as Woodville could not be omitted), attempted to give the history of the melancholy catastrophe just detailed, but without the atrocious malignity or frigid apathy of the man 'deeply imbued with medical science.' *Par parenthese,* is there then some fitness in the observations of a late flighty statesman of morbid irritability, so contrary to what was sung by the Swan of the Thames?

> 'A little learning is a dangerous thing,

> Drink deep or taste not the Pierean Spring."

>

> POPE.

> 'Nothing can be conceived more hard than the heart of a thorough-bred metaphysician. It comes nearer to the cold malignity of a wicked spirit, than to the frailties and passions of a man.'
>
> 'The Geometricians and the Chemists bring, the one from the dry bones of their diagrams, and the other from the soot of their furnaces, dispositions that make them worse than indifferent about those feelings and habitudes which are the supports of the moral world.'
>
> BURKE.

"It may have been dishonourable, cowardly, unjust, in the man 'deeply imbued in medical science,' to tread on the paws of a dead lion, which, if he had been living, might have made the trampler tremble by a shake of his mane; yet, 'deeply imbued' as is James Moore in medical science, and although Edmund Burke, whose genius he says is incontestible, might have discovered 'incorporeal, pure, unmixed, dephlegmated, defecated evil,' in his violating the sanctuary of the dead, the silence of the grave, let us hope of him that he is only the character he has taken upon himself to pronounce his Irish surgeon

'not cooled down to the medical point' to be—a character far from irreclaimable)—*Allons-mon parenthese est fini.*

"Dr. Thornton had stated and printed, that the melancholy event had happened on a clear moon-light night, as if Woodville might have seen and known who the seeming assailants of his house were at the time. But, on my informing him that, at the very time, a party of us, Cumberland youths, after a festive supper, were beating up with terriers the thickets and bushes of the hills and dales of that country, in search of those animals so destructive to the hen-roost, and that though a frosty, it was a cloudy or misty night, with snow enough on the ground, but only at a distance from the town or village, to light us through our nocturnal expedition, he cancelled the sheet, and afterwards thanked me for preserving him from obtaining the name of a detractor of the dead.

"Already, in the vindication of an old friend, I feel that *Jamque opus exegi* for which the liberal of the profession will give me credit. It will make them tolerate, perhaps, with some indulgence, the egoism of a man who, tossed about the world rather *eccentrically* nearly half a century since he first left his native hearth, is now put upon the defence of his professional character—that is, of his bread. I shall have afforded them a gratification scarcely in the power of any other individual—the gratification of seeing rescued the good name of a late estimable character from the misrepresentations of the Calumniator.

"I now conclude on this departed character, '*mourant en Socrate,*' as I heard Lafisse well express it, at a public sitting of the *Societé de Medecine de Paris,* in his *Eloge de Vicq d'Azyr,* in alluding to the patient sufferings of Baillie, Mayor of Paris, at the time of his death, yet without withholding the expression of the wish — that, when the time shall arrive, when we may wish to get rid even of the faintest recollections of the feuds and animosities, wherein we reciprocally attempted the discomfiture or harassment of the opponent, 'or ever the silver cord be loosed, or the golden bowl be broken, or the pitcher be broken at the fountain, or the wheel broken at the cistern,' when the dust shall return to the earth—the wish that our latter end may be like his.

"I conclude with presenting the information I have derived directly from the officers of the Small-pox and Inoculation Hospital.

"Dr. Adams, the successor of Dr. Woodville, as Physician to the Small-pox and Inoculation Hospital, informs me, that in conversing with him a little before his death, he complained to him of medical men addressing him as they would their own patients, even expressing hopes of his recovery, whilst they must know, that he could not be insensible of the impossibility of such event.—A few days before his decease, his friend Coleman, Professor at the Veterinary College, playing at chess with him at the hospital, the arrival of Mr. Greatrex, an undertaker, was announced. On his entering, the Doctor requested him to sit down, saying, the game was nearly finished. 'Mr. Greatrex, there was a misunderstanding between you and me in my settling with you on leaving Ely Place, and I am desirous of showing you that I am at peace with you, as I hope to die in peace with all men. I am just on the point of departing, have but very few days to live, and wish to arrange with you the order of my funeral.' The astonished undertaker had never before been so addressed; hoped the Doctor was quite mistaken, that he might yet live many years.—'Many of my friends, Mr. Greatrex, address me in this way; and were I young, I might wish a restoration to health, but the system is done—a return to health and vigour is impossible. Having been brought up a Quaker, I wish to be buried amongst them, and in their plain way, to avoid every unnecessary expence. You will therefore now consider the amount of the necessary disbursements for the occasion, and I will discharge them. It will save some trouble to my executors'."

---

*To the Editors of the Medico-Chirurgical Journal & Review.*

GENTLEMEN,

I request you would have the goodness to insert the following Notice in your Journal; which will much oblige,

Yours, &c.

W. ADAMS.

Albemarle Street, 21 March, 1818.

"Sir William Adams having had the honour to be nominated by his Majesty's Government, to superintend that part of York Hospital, Chelsea, which has been appropriated to the reception of the blind Pensioners belonging to the Army, Navy, and Artillery, feels it a duty fully to lay open to the Profession at large his new modes of treating them. This duty is suggested as well by the distinguished confidence which has been reposed in him, as by the high sanction thus conferred upon his improvements in Ophthalmic Surgery. He therefore freely invites all Medical Practitioners and Students, who are interested in the advancement of this branch of Surgery, to attend his operations at York Hospital, which, for their convenience, will be performed in future on Tuesdays and Fridays, between the hours of seven and nine in the morning.

"To remove all doubt or misconception with regard to Sir William Adams's practice, he proposes on each of these days to give a short description of the nature of one of the diseases to be operated upon; the general modes of performing the operation; his peculiar mode; and his reasons for deviating from the usual practice, where such deviation has been found necessary.

The records kept of each case from the patient's admission into hospital to his final discharge, will be open at the periods already mentioned, for the inspection of such gentlemen as attend; so that the profession will be enabled fairly to appreciate the character of the *new*, as compared with the *old* modes of practice.

"It is expected, that from *fifteen hundred to two thousand patients* will successively be placed under the care of Sir William Adams in this institution."

---

### *Case of Hydatids in the Uterus, by Dr. J. Eights, of Albany, North America.*

(From the American Medical and Philosophical Register.)

About the 20th of April, 1814, I was requested to visit Mrs. C. when she gave me the following account of her situation and complaints. She is about twenty-eight years of age, has been the mother of three living children, and has had two abortions, of which one was at the period of three months, and the other about seven months after conception. During the months of June and July, 1813, she experienced a severe attack of fever, of which she perfectly recovered by the first of August, at which time she supposed herself pregnant, and during the first four months, experienced the usual symptoms, such as nausea and vomiting, faintings, (to which she had been subject in former pregnancies,) a swelling of the breasts, and a gradual enlargement of the abdomen. When about six months advanced, she became alarmed at her condition, not having discovered any motion of the fœtus, and experiencing also a heaviness and bearing down in the lower part of the abdomen. In this situation she continued until the ensuing April, except that the heaviness increased and her health evidently declined. About the middle of this month she was seized with a discharge from the vagina of a bloody matter, which was evacuated by a sudden gush. These discharges occurred every day, and at last became so frequent, that she was seldom free from them. She had no pain except in the right side, and this was most severe at the period of the discharges. On examining the patient externally, I could not discover any inequalities or hardness in the abdomen, but a uniform surface, with the bulk of a woman when about seven months advanced in pregnancy. On examination,

*per vaginam*, the uterus was found low down in the pelvis, and the os tincæ so far opened as to admit the tip of the fore finger. The uterus, which appeared to be filled with some soft substance, was completely distended. Mrs. C. was at this time troubled with a violent cough and night sweats, and complained of a loss of appetite, and a great degree of thirst, although the quantity of urine evacuated was not less than usual. Her countenance was sunk and her spirits greatly depressed. I endeavoured to convince her of the necessity of not permitting herself to be thus affected, and flattered her with the hopes of a favourable termination, intimating at the same time my suspicion that the uterus contained hydatids.

In order to relieve the cough, I administered the common pectoral mixture, and to restore her appetite and restrain the night sweat, I gave the sulph. acid. dilut. By the use of these remedies, these symptoms were relieved, I then ordered the tinct. mur. ferr. gtt. xij, ter in die. This medicine was persisted in until the 12th of May, at which period I was again requested to visit her. She then informed me that the watery discharges had ceased for some time, but that she had discharged during the day some small clots of blood; that she had regular pains with much bearing down, and these increased in frequency; and that she felt much as in former labours, except that the pains were more rending; her abdomen had somewhat increased in bulk and hardness. On examination, I found the os uteri dilating, and ascertained that the substance in the uterus was soft and very tender, the finger meeting with little resistance.

In the evening I again saw her; the pains had become more severe, and, on introducing my hand, I found the os uteri much dilated, and it met the contents of the uterus, which, in endeavouring to bring away, broke; and on examination, I found the part thus discharged to be hydatids, attached to some pulp-like albumen. The pains continuing, she discharged about a gallon of hydatids, differing from the largeness of a pullet's egg to the size of a pea. After the discharge, she felt free from pain; there was little or no evacuation from the uterus, but about the third day she became uneasy and feverish, and shortly after had a copious secretion of milk. She is now recovering (June) as well as any woman who has been confined in child birth. Her countenance, which was considerably distorted, has become natural, her abdomen is completely reduced to its natural size, and her appetite is returning. I have directed her to continue the use of the tinct. mur. furr. both as a tonic and to destroy any remains of hydatids; being firmly convinced that this medicine has been the principal means of destroying these singular animals.

July 28. The patient is now in perfect health.

On Monday, the 9th of March 1818, the Anniversary Meeting of the Medical Society of London was held at their house, Bolt Court, when the following Officers and Members of Council were declared elected for the ensuing Year:

*President*, Dr. Walshman.—*Vice Presidents*, Dr. Merriman, Dr. Haighton, Mr. George Young, Mr. Whately.—*Treasurer*, Mr. Andree.—*Librarian*, Dr. Hancock.—*Secretaries*, Dr. Uwins, Mr. Blegborough.—*Registrar*, Mr. Pettigrew.

*Council*, Dr. Adams, Dr. Clutterbuck, Dr. Blegborough, Dr. Ley, Dr. Hooper, Dr. Fryer, Messrs. Pettigrew, Vance, Leese, R. Adams, Saner, Johnston, Sutliffe, Stevenson, Hooper, Seaton, S. Griffith, Field, Dunlop, W. Griffith, Drysdale, Robinson, Radford, Hilliard, Brown, T. Bartlett, Brougham, Newby, Winter, Wadd.

To deliver the Anniversary ORATION 1819, Mr. Whately.

The Oration was by Dr. Uwins, and consisted of a statement of the Orator's sentiments on the present condition of Medical Science and Practice. Dr. U. commenced his discourse by deprecating the charge of dogmatism, which might attach to a faithful discharge of his duty, and then briefly proceeded to defend modern medicine against the allegations of those who denied the certainty of the science, and the assumed improvement in the practice of the Healing Art. Having discussed this preliminary matter, the Orator entered upon the more immediate business of his discourse, and divided the prevailing doctrines of the day into, first, those which too exclusively regard the liver as the prime source of every morbid derangement; secondly, such as devote their whole attention to the condition of the digestive organs and functions; and, lastly, those views which, in opposition to the stimulating principles of the Edinburgh Schools, contemplate every thing in the way of disorder as constituted by congestive and inflammatory states. Upon the *pro* and *con* of each of these pathological postulata, Dr. U. took occasion to expatiate; and he seemed, although severe upon systematic extravagance, to wish that his auditors should be impressed with the conviction, that the several doctrines upon which he took occasion to animadvert were not, in his mind, objectionable in any other light than as being too generalizing in their structure, and too excluding of other considerations in their practical application. He concluded his harangue by expressing the obligations which the medical world is under to the several Systematics to whom he had more especially alluded, and by cautioning his hearers against attachment to any one set of opinions or systems of pathology and practice. The room was crouded, and we were gratified in observing that the auditors were not confined to the Society's own members.

---

At the Anniversary Meeting of the Lord Mayor, Aldermen, and other Governors of the City Hospitals, at Christ Church,

Newgate Street, on Easter Monday last, the following Statement was read, after a Sermon preached on the occasion by the Bishop of Llandaff, viz.

CHRIST'S HOSPITAL.

| | |
|---|---|
| Children put forth Apprentices - - - - - - - - - - | 157 |
| Buried last year - - - - - - - - - - - - - - - - - | 13 |
| Children now under care of the Hospital - - - - - - | 1196 |
| To be admitted on presentation - - - - - - - - - - | 130 |
| | 1496 |

ST. BARTHOLOMEW'S HOSPITAL.

| | |
|---|---|
| Patients admitted, cured, and discharged last year— | |
| In-patients - - - - - - - - - - - - - - - - - | 2599 |
| Out-patients - - - - - - - - - - - - - - - - | 5607 |
| Buried last year - - - - - - - - - - - - - - - - | 195 |
| Remaining under cure—In-patients - - - - - - - - | 350 |
| Out-patients - - - - - - - | 300 |
| So that there have been under the care of this Hospital last year - - - - - - - - - - - - - - - - - | 9051 |

ST. THOMAS'S HOSPITAL.

| | |
|---|---|
| Cured and discharged last year - - - - - - - - - - | 9165 |
| Remaining under cure—In-patients - - - - - - - - | 395 |
| Out-patients - - - - - - - | 316 |
| Buried last year - - - - - - - - - - - - - - - - - | 170 |
| | 10046 |

BRIDEWELL HOSPITAL.

| | |
|---|---|
| Vagrants committed by the Lord Mayor and Sitting Aldermen - - - - - - - - - - - - - - - - - | 244 |
| Apprentices sent for solitary confinement - - - - - - | 28 |
| Persons to be sent to different Parishes - - - - - - - | 2021 |
| Apprentices to be brought up in different trades - - - | 30 |
| | 2323 |

BETHLEM HOSPITAL.

| | |
|---|---|
| Remaining in the Hospital - - - - - - - - - - - - - | 309 |
| Buried last year - - - - - - - - - - - - - - - - - | 4 |
| Cured and discharged last year - - - - - - - - - - | 142 |
| Patients under cure - - - - - - - - - - - - - - - | 196 |
| Ditto incurables - - - - - - - - - - - - - - - - - | 65 |
| | 716 |

### NEW BOOKS.

Observations on Phagedæna Gangrænosa, by H. Home Blackadder, 8vo.

A new Volume on the Diseases of the Eye, by the late Mr. Ware, 8vo.

Mr. S. F. Gray, Apothecary and Teacher of Botany and Materia Medica has in the press, and nearly ready, a work intended to serve as a Supplement to the several Pharmacopœias, containing the medical uses of all such Plants as have been hitherto examined, and an arrangement of their uses; a glossary of the terms and contractions used by Physicians in their prescriptions; usual medical formulæ arranged in classes; botanical practice of medicine, offered as hints to regular practitioners to improve the art.—This work is meant to supply the deficiencies of the present College Pharmacopœias, which being merely intended to direct the preparation of the medicines most usually employed by the regular practitioners, are of course defective in regard to the very numerous articles that are kept in the retail shops to supply their other customers, as medicines, perfumery, liqueurs, sauces, British wines, paints, varnishes, &c.

---

Died. At Oxford, aged nearly 80, Mr. Richard Rawlins, Surgeon, Accoucheur, and Apothecary.—In Mabledon Place, New Road, Mr. Peter Matthias, Surgeon.—At Sydenham, aged 82, Hugh French, M. D.

